# Cost-effectiveness and sustainability of improved hospital oxygen systems in Nigeria

**DOI:** 10.1136/bmjgh-2022-009278

**Published:** 2022-08-10

**Authors:** Hamish R Graham, Ayobami A Bakare, Adejumoke Idowu Ayede, Joseph Eleyinmi, Oyaniyi Olatunde, Oluwabunmi R Bakare, Blessing Edunwale, Eleanor F G Neal, Shamim Qazi, Barbara McPake, David Peel, Amy Z Gray, Trevor Duke, Adegoke G Falade

**Affiliations:** 1 Centre for International Child Health, University of Melbourne, MCRI, The Royal Children's Hospital, Parkville, Victoria, Australia; 2 Department of Paediatrics, University College Hospital Ibadan, Ibadan, Nigeria; 3 Department of Community Medicine, University College Hospital Ibadan, Ibadan, Nigeria; 4 Global Public Health, Karolinska Institute, Stockholm, Sweden; 5 Department of Paediatrics, School of Medicine, University of Ibadan, Ibadan, Nigeria; 6 Infection and Immunity, Murdoch Childrens Research Institute, Parkville, Victoria, Australia; 7 Independent Consultant Paediatrician, Geneva, Switzerland; 8 Nossal Institute for Global Health, Melbourne, Victoria, Australia; 9 Ashdown Consultants, Hartfield, UK

**Keywords:** Child health, Health economics, Health services research, Paediatrics, Pneumonia

## Abstract

**Introduction:**

Improving hospital oxygen systems can improve quality of care and reduce mortality for children, but we lack data on cost-effectiveness or sustainability. This study evaluated medium-term sustainability and cost-effectiveness of the Nigeria Oxygen Implementation programme.

**Methods:**

Prospective follow-up of a stepped-wedge trial involving 12 secondary-level hospitals. Cross-sectional facility assessment, clinical audit (January–March 2021), summary admission data (January 2018–December 2020), programme cost data. Intervention: pulse oximetry introduction followed by solar-powered oxygen system installation with clinical and technical training and support. Primary outcomes: (i) proportion of children screened with pulse oximetry; (ii) proportion of hypoxaemic (SpO_2_ <90%) children who received oxygen. Comparison across three time periods: preintervention (2014–2015), intervention (2016–2017) and follow-up (2018–2020) using mixed-effects logistic regression. Calculated cost-effectiveness of the intervention on child pneumonia mortality using programme costs, recorded deaths and estimated counterfactual deaths using effectiveness estimates from our effectiveness study. Reported cost-effectiveness over the original 2-year intervention period (2016–2017) and extrapolated over 5 years (2016–2020).

**Results:**

Pulse oximetry coverage for neonates and children remained high during follow-up (83% and 81%) compared with full oxygen system period (94% and 92%) and preintervention (3.9% and 2.9%). Oxygen coverage for hypoxaemic neonates/children was similarly high (94%/88%) compared with full oxygen system period (90%/82%). Functional oxygen sources were present in 11/12 (92%) paediatric areas and all (8/8) neonatal areas; three-quarters (15/20) of wards had a functional oximeter. Of 32 concentrators deployed, 23/32 (72%) passed technical testing and usage was high (median 10 797 hours). Estimated 5-year cost-effectiveness US$86 per patient treated, $2694–4382 per life saved and $82–125 per disability-adjusted life year-averted. We identified practical issues for hospitals and Ministries of Health wishing to adapt and scale up pulse oximetry and oxygen.

**Conclusion:**

Hospital-level improvements to oxygen and pulse oximetry systems in Nigerian hospitals have been sustained over the medium-term and are a highly cost-effective child pneumonia intervention.

**WHAT IS ALREADY KNOWN ON THIS TOPIC**

- Oxygen and pulse oximetry are critical hospital services poorly available to many populations, particularly in poor and rural communities.
- Improving hospital oxygen services can improve quality of care processes and reduce in-hospital mortality from pneumonia among young children.
- We know little about the cost-effectiveness of improved hospital oxygen systems or whether clinical and technical practice outcomes can be sustained in the medium-term or long-term.

**WHAT THIS STUDY ADDS**

- Pulse oximetry and oxygen practices were maintained at high levels after direct programme support had been withdrawn; however, some hospitals struggled more than others.
- Hospital-level improvements to pulse oximetry and oxygen services were highly cost-effective, comparing favourably to essential child health interventions such as immunisation.
- Further improvements to oxygen systems effectiveness, sustainability and affordability may be possible with sustained support for technicians and integration of new technology.

**HOW THIS STUDY MIGHT AFFECT RESEARCH, PRACTICE OR POLICY**

- Improving district hospital pulse oximetry and oxygen services is feasible and cost-effective over the medium-term and should be a policy priority.
- Health facility leaders and policymakers can use our practical lessons to sustain healthcare worker care practices, enhance biomedical technician capacity and equipment lifespan and integrate solar power solutions.
- Future oxygen systems research should aim to measure the sustainability of practice change and equipment function over the medium-term to longer-term, and their determinants and include robust cost-effectiveness analysis.

## Introduction

Oxygen therapy is a standard of care for treating hypoxaemia (low blood oxygen) and respiratory compromise,^1 2^ and is required for approximately 10%–15% of children (including 31% of children with pneumonia) and 20% of neonates admitted to hospital globally.^3 4^ Oxygen services are essential for every health facility that admits children, including routine pulse oximetry to identify hypoxaemia, reliable oxygen supplies and adequate delivery equipment and healthcare worker capacity.^5 6^

Patients needing oxygen depend on prompt recognition of hypoxaemia using pulse oximetry, reliable availability of oxygen at the point of care and provision of services at an acceptable cost.^7^ We know that pulse oximetry and oxygen practices are severely inadequate in many low-resource contexts (particularly in smaller hospitals and health facilities where the majority of patients present) and can be improved in the short-term with education and supplies.^7–14^ Limited data suggest that oxygen concentrator-based systems can function for many years with routine basic maintenance and access to repairs.^15 16^ However, broken equipment is ubiquitous in many low-resource settings raising questions of how maintenance practices can be improved and sustained.^17–19^ We know that oxygen equipment and supplies are major costs for health systems, individual facilities and patients,^7 17 20 21^ but there are few data to support cost-effectiveness analyses or decisions.^22–24^

In 2015, we commenced the Nigeria Oxygen Implementation project to improve hospital oxygen systems in 12 hospitals in southwest Nigeria. We conducted an unblinded stepped-wedge trial comparing preintervention (usual care) to the introduction of pulse oximetry followed by the introduction of a multifaceted, concentrator-based oxygen system.^25^ Results showed improvement in pulse oximetry coverage for acutely unwell children (an increase from 4% to 92%) and oxygen provision to those with hypoxaemia (an increase from 74% to 82%), with the largest change occurring in the first few months after the introduction of pulse oximetry.^26^ We found a reduction in the risk of death for children (aged <15 years excluding neonates) admitted with pneumonia following the introduction of pulse oximetry (aOR 0.33, 95% CI 0.12 to 0.92) and full improved oxygen system (aOR 0.50, 0.26 to 0.98), with no difference between pulse oximetry and full improved oxygen system periods (aOR 1.09, 0.50 to 2.41).^13 26^ We found no difference in all-cause child mortality from pulse oximetry introduction (aOR 0.97, 0.60 to 1.58) or the full oxygen system (aOR 1.03, 0.72 to 1.47).^26^ We found discordant results for neonates with no mortality benefit from pulse oximetry introduction (aOR 0.90, 0.57 to 1.43) or full improved oxygen system (aOR0.90, 0.62 to 1.24) but relative increased risk of death for neonates comparing the full oxygen system to pulse oximetry period (aOR 1.45, 1.04 to 2.00) thought to be related to factors external to the intervention.^26^

A recent meta-analysis that included additional studies from small and medium-sized health facilities in the Asia-Pacific region suggests that improvements in hospital oxygen systems can reduce the risk of death for admitted children by approximately 20%, with particular benefit for children with pneumonia (50% reduction in risk of death).^24^

So, while there is short-term evidence showing the benefits of improved hospital oxygen systems for children, few studies have reported medium-term cost-effectiveness or sustainability of technical and clinical practice outcomes.

This study aimed to provide medium-term evidence on oxygen availability, oxygen-related care practices, clinical outcomes, programme costs and cost-effectiveness from the 12 facilities involved in the Nigeria Oxygen Implementation project.

## Methods

We conducted a prospective evaluation of the sustainability and effectiveness of improved oxygen systems in 12 hospitals as part of a mixed-methods evaluation of the Nigeria Oxygen Implementation project. It involved cross-sectional facility assessments and clinical audits performed 5 years after introducing pulse oximetry and improved oxygen systems. Detailed methods of our stepped wedge trial have been reported previously.^25 26^

### Participants and context

We conducted our study in 12 secondary health facilities in four states (Oyo, Ondo, Osun and Ogun) in south-west Nigeria. Nigeria is a populous lower-middle-income country with high child mortality rates (117.2 per 1000 live births in 2019), and pneumonia is the leading cause of death.^27^ Secondary health facilities are intended to be first-line admission facilities with a mix of public, private for-profit and private not-for-profit (typically religious mission) providers. We selected a mix of government and private non-profit (mission) hospitals of varying sizes, intended to be representative of secondary health facilities that admitted children (described in detail elsewhere).^25^ We focused our work in the paediatric and neonatal units of participating hospitals.

During the early intervention period, and extending for the next 4 years (2016–2020), Nigeria plunged into a nationwide recession that impacted negatively on health facilities, staff and the general population.^28^ The recession added to existing health workforce challenges precipitating closure of some of the participating government hospitals for weeks to months due to industrial action over unpaid wages and likely affected care-seeking and staff motivation. Nigeria also experienced the global COVID-19 pandemic, with the Nigerian Centre for Disease Control (NCDC) reporting peaks in major cities in mid-2020, early-2021 and late-2021.^29^

### Intervention

We introduced pulse oximetry to the paediatric and neonatal areas of all 12 hospitals in October–November 2015. We provided handheld pulse oximeters (Lifebox Foundation), conducted short (~1 hour) task-based pulse oximetry training for nursing and medical staff based on WHO guidelines,^2 30^ and distributed a short instructional video.^31^ Over the subsequent 16 months, hospitals received additional training and an improved oxygen delivery system according to a prespecified randomisation order (clusters of 3 hospitals, every 4 months). The improved oxygen delivery system involved the installation of oxygen concentrators (1–4 per facility) and simple distribution systems (flow-splitters and plastic) enabling individual titration of oxygen to multiple patients from a single oxygen source. We conducted training on oxygen equipment for technicians (3-day central workshop) provided equipment checklists and essential tools (eg, oxygen analyser) where needed. We trained a small group of local nurses and doctors at each site on the clinical use of oxygen and participatory teaching methods, then supervised them to train all their colleagues through a series of half-day, onsite, practical workshops.^25^ We encouraged these trainers to conduct retraining as new staff rotated through the wards, but we did not organise formal refresher training.

Following initial set-up and training, the project team provided 2 years of intensive support, gradually reducing until formal handover in 2017. During the intensive support period, the project team conducted quarterly supervisory visits and communicated regularly regarding the ongoing collection of clinical data and equipment function. A dedicated research nurse at each site coordinated clinical data collection and management and was available for on the ground support or troubleshooting. Project biomedical engineers (BMEs) were located at a central site (University College Hospital, Ibadan) and were available for troubleshooting over the phone and in-person maintenance and repairs. Initially, the central BMEs supervised local technicians in preventive maintenance, reducing contact as local technicians developed confidence. The central BMEs remained available throughout the programme for major faults and repairs, providing a swap-and-go service and performing most repairs at the central workshop where spare parts and tools were stored. We encouraged hospitals to develop local multidisciplinary quality improvement teams involving technicians, healthcare workers and management personnel to establish and sustain oxygen maintenance and clinical care practices and systems.

Prior to formal handover, it was clear that the local biomedical capacity to perform repairs remained low, with limited access to spare parts and tools and minimal support from government agencies. Members of the project team formed a non-profit oxygen service organisation (Oxygen for Life Initiative) to continue providing support to the original 12 hospitals and expand services to other facilities. Under this new arrangement, hospitals bore responsibility for routine maintenance and would contact the Oxygen for Life Initiative BME for technical assistance, including site visits, spare parts and repairs as required (provided free or at cost).

### Procedures

We obtained data for this study from multiple sources using tools and data collection methods that were the same or very similar to what we had used for our previous baseline and programme evaluation.^17 25 26 32^ We obtained summary clinical data on ward admissions and outcomes from the ward register monthly over 3 years (2018–2020). We obtained deidentified clinical data from a retrospective clinical audit of all children and neonates admitted over 3 months (January–March 2021), including patient age, presenting symptoms/signs (including SpO_2_ if documented), diagnosis, and oxygen use.

We obtained data on health facility service capacity using our standardised health facility assessment form (adapted from previously used WHO facility assessment tools),^16–18^ through direct observation and discussion with managerial, clinical and technical staff. This included data on staffing, bed capacity, oxygen supply, oximeter supply, guidelines/protocols, power supply and costs. We tested concentrators and oximeters using our standardised tool, including assessing the oxygen purity of the concentrator’s gas with calibrated oxygen analysers (Maxtec, Salt Lake City, USA) and oximeter function with ProSim SPOT Light (Fluke Corporation, Everett WA, USA). We also recorded informal feedback from technicians and hospital staff during field visits.

Trained nurses and biomedical engineers collected these data during facility visits between May and September 2021. Data collectors recorded the data on paper forms, then input the data into ODK software. AB checked completed databases for completeness, then transferred the data files to HRG for cleaning and analysis and clarified gaps with data collection staff where required.

### Outcomes

We evaluated clinical practice outcome data for all children and neonates admitted during the audit period and focused cost-effectiveness analysis on the subgroup of children with pneumonia for whom we observed mortality benefit.^26^ We approached costs from a facility/service provider perspective.

Our primary outcomes were (i) the proportion of children and neonates who were screened with pulse oximetry and (ii) the proportion of children and neonates with hypoxaemia (SpO_2_<90%) who received oxygen therapy. These outcomes were selected to be consistent with our primary stepped wedge trial analysis,^26^ and reflective of a patient-centred approach to measuring oxygen access.^7^

Secondary outcomes included overall oxygen use; appropriateness of oxygen use relative to clinical signs; death (defined as death during hospitalisation or discharged expected to die); the proportion of wards with functional oxygen source; the proportion of project concentrators and oximeters still available and functional. Economic outcomes included total programme costs; costs per facility; cost per life saved; cost per disability-adjusted life year (DALY) averted.

We did not calculate a prespecified sample size for the primary practice outcomes and decided that 2–3 months of clinical practice data would reasonably represent current practices and enable meaningful comparison to previous periods.

We used WHO case definitions to classify diagnoses using clinical signs documented in the patient chart.^2^ Neonatal diagnostic classifications (neonatal sepsis, jaundice and neonatal encephalopathy) were classified according to the admission diagnosis.

### Analysis

We used Stata V.17 (StataCorp, College Station, USA) for data cleaning and analysis.

We present results according to the three key domains of oxygen access: oxygen availability, oxygen use and cost.^7^ We used summary statistics and charts to present data on facility oxygen capacity, equipment functionality over time and patient oxygen cost.

We used summary statistics and charts to visually depict practice change over time, comparing the follow-up with preintervention and intervention periods. We used mixed-effects logistic regression to compare outcomes in the follow-up period to previous study periods, using the same model set-up as previous.^26^ This analytical model included random effects to adjust for clustering at the hospital level and fixed effects for intervention and time (4-month steps), including hospital-time interaction (see details reported previously).^26^

We calculated the cost-effectiveness of the intervention using documented programme implementation costs, recorded deaths, calculating counterfactual deaths using the relative risk estimates from our effectiveness study.^26^ Programme costs were categorised as equipment costs (including freight and customs), implementation costs (installation, training, maintenance and support) and solar costs, excluding the research costs of the programme. We report costs in US$ as this was the currency of expenditure for the high-cost equipment items and the currency of the funder, converting local spending from Naira to US$ using the average rate of exchange in 2016 (315:1). To calculate the number of pneumonia deaths during the follow-up period, we used documented admission and death numbers and assumed that pneumonia accounted for the same proportion of deaths during the follow-up period as during the earlier postintervention period (21.7%).^26^ To calculate the number of patients receiving oxygen during the follow-up period, we used documented admission numbers with oxygen administration rates from the 3-month follow-up period. We calculated conservative cost-effectiveness estimates by restricting analysis to the original data from our effectiveness study,^26^ thus reflecting the impact achieved during the 2-year postintervention period (pulse oximetry and full oxygen system periods). We calculated best-case and mid-range cost-effectiveness estimates by extending the impact over the additional 3-year follow-up period modelling for full (100%) and attenuated (50%) effect. We calculated the cost per life saved and converted this to the cost per DALY averted using a multiplication factor of 33 (the number of DALYs lost due to a death in infancy).^33^ We present cost-effectiveness estimates with and without the solar costs, recognising that solar was a power fix supplemental to the core intervention and that alternate (more cost-efficient) power fixes could have been substituted.

We followed recommended reporting guidelines for quality improvement (SQUIRE V.2.0)^34^ and economic studies (CHEERS).^35^

### Ethics

Our study was approved by the University of Melbourne Health Sciences Human Ethics Sub-Committee (1647681) and the University of Ibadan/University College Hospital Ethics Committee (16/0445).

### Role of the funding source

The funding agency had no role in the planning, conduct or analysis of this study.

### Patient or participant involvement

Patients were not involved in the design or conduct of this study. Hospital staff participated in programme design and implementation but were not involved in the analysis. Our reflexivity statement describes how the participating research partners collaborated and how this research fitted with local priorities (online supplemental reflexivity statement in the online Supplemental material).

10.1136/bmjgh-2022-009278.supp1 Supplementary data

## Results

We successfully obtained clinical, administrative and equipment data from all 12 hospitals participating in the Nigeria Oxygen Implementation programme.

### Facility characteristics

Health facility assessments showed that hospitals provided similar paediatric and neonatal services in 2021 as 2015, with some reduction in neonatal services at two larger hospitals (H7 and H10) (online supplemental tables S1 and S2 in the online Supplemental material).

10.1136/bmjgh-2022-009278.supp2 Supplementary data

We obtained summary admission and death data for 2018 to 2020 from 10 hospitals but could not locate admission books at the remaining two small rural facilities (H2 and H9). Excluding these, the mean number of children admitted to participating hospitals each month was 7% higher during the 3-year follow-up period (717, range 24–186) than the preceding 2-year intervention period (668, range 23–120), with two large hospitals increasing admissions by >30% (H1 and H7) (online supplemental table S3 in the online supplemental material). Conversely, the mean number of monthly child deaths and overall case fatality rate (CFR) was lower during the follow-up period (22.3, range 0.6–5.5, CFR 3.1%) than the intervention period (27.6, range 1.3–2.9, CFR 4.1%).

### Oxygen and pulse oximetry practices

The population captured in the 3-month clinical follow-up data were broadly similar to those in the preintervention and immediate postintervention periods, with slight variation in age and small differences in some diagnostic categories (table 1).

**Table 1:** Population characteristics across study periods

	Preintervention	Pulse oximetry only	Full oxygen system	Follow-up
	(n=24 117)	(n=10 267)	(n=14 592)	(n=1020)
Neonate (<28 days)	8813 (36.9%)	2983 (29.2%)	4733 (32.6%)	392 (38.6%)
Infant (1–12 months)	4709 (19.7%)	2218 (21.7%)	2520 (17.4%)	221 (21.8%)
Young child (1–5 years)	7788 (32.6%)	3580 (35.0%)	4803 (33.1%)	269 (26.5%)
Older child (5–15 years)	2559 (10.7%)	1452 (14.2%)	2458 (16.9%)	134 (13.2%)
Age, months median (IQR)	9.0 (0.1–24.0)	11.1 (0.3–31.0)	12.0 (0.1–36.0)	7.0 (0.1–24.0)
Age, months mean (SD)	21.0 (32.2)	24.8 (35.7)	27.0 (37.2)	22.2 (36.4)
Sex, % female	43.9	43.2	44.2	45
Hospital type, % government	57.7	62.4	57.6	54.5
Hospital size, % small	11.9	16.8	14.2	17.8
Child diagnoses and presenting signs				
Pneumonia	1897 (12.4%)	883 (12.1%)	1269 (12.9%)	76 (12.1%)
Malaria	5230 (34.3%)	2609 (35.9%)	3835 (38.9%)	214 (34.1%)
Diarrhoea	1732 (11.3%)	1053 (14.5%)	1013 (10.3%)	158 (25.2%)
Malnutrition	237 (1.7%)	184 (2.6%)	192 (2.0%)	6 (1.0%)
HIV-infected	18 (0.1%)	10 (0.1%)	13 (0.1%)	2 (0.3%)
Fever	10 005 (78.7%)	5675 (80.6%)	8022 (81.6%)	473 (75.3%)
Cough or difficulty breathing	3670 (28.9%)	1866 (26.5%)	2560 (26.0%)	165 (26.3%)
Any WHO emergency sign	4489 (35.2%)	2530 (35.9%)	3259 (33.1%)	226 (36.0%)
Hypoxaemia (SpO_2_<90%)	*46/500* (*9.2%*)	424/4274 (9.9%)	928/9057 (10.3%)	57/506 (11.3%)
Neonatal diagnoses and presenting signs				
Small/preterm	1883 (26.8%)	688 (26.4%)	1137 (26.7%)	102 (27.3%)
Neonatal sepsis	3916 (48.1%)	1705 (59.4%)	2297 (51.9%)	228 (58.2%)
Neonatal encephalopathy	3439 (42.3%)	1071 (37.3%)	1882 (42.6%)	112 (28.6%)
Jaundice	2001 (24.6%)	799 (27.8%)	938 (21.2%)	106 (27.0%)
Any WHO emergency sign	1206 (15.2%)	584 (20.1%)	887 (18.7%)	50 (12.8%)
Hypoxaemia (SpO_2_<90%)	*30/189* (*13.7%*)	425/1474 (22.4%)	993/3479 (22.2%)	53/326 (16.3%)

**Figure 1:**
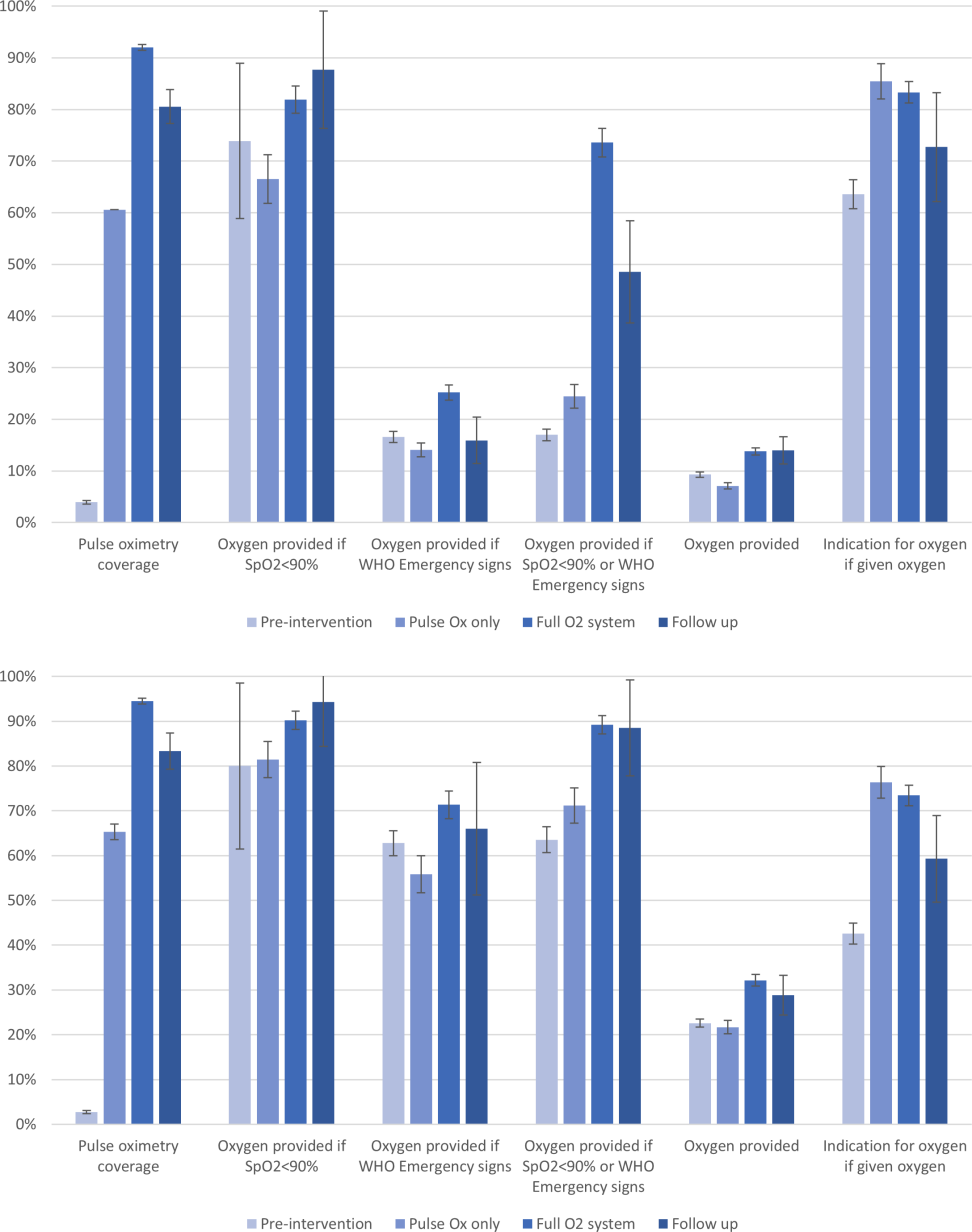
Oxygen-related clinical practice change among children (top) and neonates (bottom) admitted to 12 hospitals in southwest Nigeria. Pulse oximetry coverage and oxygen provision expressed as the proportion of all children/neonates unless otherwise specified. ‘Indication for oxygen’ is expressed as the proportion of those given oxygen who had SpO_2_<90% or WHO emergency signs. Error bars showing 95% CIs. Note: the oxygen coverage to patients with hypoxaemia indicator is biased in the preintervention period due to extremely low pulse oximetry coverage.

Overall, the improvements observed in pulse oximetry and oxygen practices during the intervention periods were sustained in the follow-up period (figure 1, table 2).

**Table 2:** Practice outcomes across preintervention, pulse oximetry, full oxygen system and follow-up periods

	Preintervention	Pulse ox only	Full O_2_ system	Follow-up	P value*
	(n=24 117)	(n=10 267)	(n=14 592)	(n=1020)	
Child practice outcomes					
Pulse oximetry coverage	500/12737 (3.9%)	4274/7055 (60.6%)	9057/9840 (92.0%)	506/628 (80.6%)	<0.001
Oxygen if SpO_2_<90%	34/46 (73.9%)	282/424 (66.5%)	760/928 (81.9%)	50/57 (87.7%)	0.262
Oxygen if WHO emergency signs	745/4489 (16.6%)	356/2530 (14.1%)	821/3259 (25.2%)	36/226 (15.9%)	0.002
Oxygen if SpO_2_<90% (or WHO emergency signs if SpO_2_ missing)	699/4113 (17.0%)	331/1354 (24.5%)	778/1057 (73.6%)	51/105 (48.6%)	<0.001
Oxygen	1184/12737 (9.3%)	502/7055 (7.1%)	1356/9840 (13.8%)	88/628 (14.0%)	0.173
Indication for oxygen†	753/1184 (63.6%)	429/502 (85.5%)	1130/1356 (83.3%)	64/88 (72.7%)	0.11
Mean starting flow rate (SD)	1.8 (0.9)	1.6 (0.9)	1.3 (0.6)	1.8 (0.7)	<0.001
Neonatal practice outcomes					
Pulse oximetry coverage	219/7940 (2.8%)	1899/2907 (65.3%)	4472/4732 (94.5%)	326/392 (83.2%)	<0.001
Oxygen if SpO_2_<90%	24/30 (80.0%)	346/425 (81.4%)	896/993 (90.2%)	50/53 (94.3%)	0.322
Oxygen if WHO emergency signs	757/1206 (62.8%)	326/584 (55.8%)	633/887 (71.4%)	33/50 (66.0%)	0.416
Oxygen if SpO_2_<90% (or WHO emergency signs if SpO_2_ missing)	712/1121 (63.5%)	403/566 (71.2%)	911/1021 (89.2%)	54/61 (88.5%)	0.864
Oxygen	1794/7940 (22.6%)	631/2907 (21.7%)	1522/4732 (32.2%)	113/392 (28.8%)	0.87
Indication for oxygen†	764/1794 (42.6%)	482/631 (76.4%)	1118/1522 (73.5%)	67/113 (59.3%)	0.001
Mean starting flow rate, LPM (SD)	1.3 (0.6)	1.1 (0.5)	0.9 (0.4)	1.0 (0.5)	0.027

See online supplemental table S4 in online supplemental material for the full mixed-effects regression model. Data are *n*/*N* (%) unless otherwise indicated. Denominators vary according to the population included. Pulse oximetry and oxygen coverage expressed as the proportion of all children/neonates unless otherwise specified. Note: the oxygen coverage to patients with hypoxaemia indicator is biased in the preintervention period due to extremely low pulse oximetry coverage.

*P values for the test of difference between follow-up and full oxygen system periods using Student’s t-test for means and Pearson’s χ^2^ test for proportions.

†‘Indication for oxygen’ is calculated as (# with SpO_2_<90% or WHO emergency signs on admission)/# prescribed oxygen therapy). This outcome measure may be biased given the dramatic improvement in SpO_2_ documentation after the preintervention period.

LPM, litres per minute.

Pulse oximetry coverage for both neonates and children remained high during the follow-up period (83% and 81%, respectively), dropping a little from the coverage achieved during the full oxygen system period 3 years prior (94% and 92%) and remaining much higher than during the preintervention period (3.9% and 2.9%) (figure 1, table 2, online supplemental tables S4 in online supplemental material). Oxygen coverage for hypoxaemic neonates and children was similarly high (94% and 88%), marginally higher than the full oxygen system period (90% and 82%).

Heat map depiction of these practice changes showed some variation between facilities, with the notable deterioration in oxygen-related practices in a small minority of facilities (online supplemental figure S1 in online supplemental material). One large government hospital that exclusively served women and children in a major city (H7) ceased using pulse oximetry altogether and stopped offering oxygen services on the paediatric ward. Two small facilities in a rural area (H2 and H9) used pulse oximetry on a minority of patients and rarely provided oxygen therapy.

Secondary practice outcomes showed that 29% of neonates and 14% of children were administered oxygen during the follow-up period—a similar usage rate to the full oxygen system period (32% and 14%) and higher than before the improved oxygen supplies were installed (23% and 9%) (figure 1, table 2, online supplemental table S4 in online supplemental material). Of those given oxygen, fewer neonates (59% vs 75%) and children (73% vs 85%) had a clear indication for oxygen (ie, hypoxaemia or WHO emergency signs) in the follow-up period compared with the full oxygen system period, suggesting that HCWs were providing oxygen more liberally to patients without hypoxaemia.

### Oxygen and pulse oximetry equipment and supply

Functional oxygen sources (oxygen concentrator or cylinder) were present in 11/12 (92%) paediatric areas and all (8/8) neonatal areas—the notable exception being the paediatric ward of H7, which had relocated the project concentrators to the Emergency Department and no longer offered oxygen services on the ward (table 3). Three-quarters (15/20) of ward areas had a functional oximeter—mostly fingertip devices that had replaced the original project handheld devices (eight original project oximeters were located, of which five were still working). Most facilities (9/12, 75%) were still using the oxygen delivery points installed by the programme and 12/20 (60%) still displayed the oxygen guideline (wall-chart).

**Table 3:** Oxygen equipment availability and functionality on paediatric and neonatal wards in 12 hospitals in southwest Nigeria, 2021

Hospital ID	SHHL	BMC Saki	SH Abeokuta	SSH Akure	OMCH	SDAHI	MCH Akure	Adeoyo	SH Saki	Oluyoro	OLFCH	SH Oyo
	H1	H2	H3	H4	H5	H6	H7	H8	H9	H10	H11	H12
Paediatric ward												
Functional oxygen source	Yes	Yes	Yes	Yes	Yes	Yes	No	Yes	Yes	Yes	Yes	Yes
Cylinders functional*	1/1	2/2	1/1	1/1*	0	1/1*	0	1/1	4/4*	Shared	5/5	0/1
Concentrators functional†	3/3	0/1	1/2	1/1§	1/1	0¶	0**	2/2	0/1	2/2	1/1	1/1
Pulse oximeter functional‡	1/1	1/2	1/2	2/2	1/1	1/1	0	1/3	1/2	Shared	2/2	0/1
Oxygen delivery points	10	5	10	5	5	0	0	5	5	7	5	1
Oxygen delivery devices	Yes	Yes	Yes	No	Yes	No	No	Yes	Yes	Yes	Yes	Yes
Oxygen guideline	Yes	Yes	Yes	No	No	No	No	No	Yes	Yes	Yes	Yes
Neonatal ward												
Functional oxygen source	Yes	Yes	Yes	Yes	Yes	–	Yes	Yes	–	Yes	–	–
Cylinders functional*	1/1	2/2	Shared	3/3	1/1	–	0	1/1	–	1/1	–	–
Concentrators functional†	3/3	1/1	Shared	2/2	1/1	–	2/2	1/1	–	1/1	–	–
Pulse oximeter functional‡	1/1	1/2	Shared	2/2	1/1	–	0	1/1	–	1/1	–	–
Oxygen delivery points	10	3	5	5	5	–	9	5	–	5	–	–
Oxygen delivery devices	Yes	Yes	Yes	Yes	Yes	–	Yes	Yes	–	Yes	–	–
Oxygen guideline	Yes	Yes	No	No	Yes	–	No	Yes	–	Yes	–	–

*Cylinder functionality defined as equipped with appropriate regulator apparatus and at least partially full (* if cylinder is primary oxygen source)

†Concentrator functionality defined as able to produce minimum 83% oxygen purity at 5 litre per minute flow.

‡Pulse oximeter functionality defined as able to turn on and produce a reading on the tester’s finger (NB: most oximeters were not able to be more comprehensively tested with an oximeter simulation device).

§Temporarily not being used due to faulty flowmeter distribution awaiting repair.

¶Project concentrator lost.

**Project concentrators relocated to the emergency department.

We originally purchased 38 concentrators of which 29 were directly deployed to participating hospitals and 3 were deployed to replace broken concentrators, 1 was faulty on delivery and only ever used for training purposes and 5 proved surplus to need and were deployed elsewhere (n=3) or retained in store (n=2). Of the 32 concentrators ever deployed in participating hospitals, 23 (72%) were fully functional, 3 (9%) were broken and retired from service, 2 (6%) were unable to be located and 4 (13%) failed testing due to low oxygen purity (online supplemental table S5 online supplemental material). Concentrators had a mean usage of 12 913 hours (median 10 797 hours, IQR 5457 to 21281)—equivalent to 1.5 years non-stop usage.

### Costs

#### Patient fees

All hospitals charged patients for oxygen services. The median cost charged to patients for 1 day of oxygen therapy was 2000 Naira (IQR N1000–5000) (USD$5.26, IQR $2.63–13.16)—similar to the preintervention period (online supplemental tables S1 and S2 online supplemental material).

#### Programme costs

The programme costs of oxygen equipment, training and support were modest (mean USD$19 530 per facility), dwarfed by the cost of solar power (mean USD$55 447 per facility) (online supplemental tables S6 online supplemental material). Solar power costs were substantially higher than anticipated, as failure of the first cluster of solar power installations led to a decision to substantially increase the capacity of subsequent installation. In retrospect, alternative power solutions may have been adequate for some facilities.

#### Cost-effectiveness

##### Per patient treated

During the intervention periods, 4686 neonates and children received oxygen therapy at a cost of USD$192 per patient treated (USD$50 excluding solar costs). Extrapolating over the additional 3 years, we estimate that an additional 5406 patients received oxygen, reducing the 5-year cost per patient treated to $89 ($23 excluding solar).

##### Per child’s life saved from pneumonia death, DALYs-averted

During the original 2-year intervention period, we recorded 141 deaths from pneumonia among children under 15 years of age. We estimated 141 deaths averted (effect size 0.5 across pulse oximetry and full oxygen system period compared with preintervention period), at a cost of US$6381 per life saved ($1662 excluding solar) and US$193 per DALY-averted ($50 excluding solar) (table 4). Extrapolating to include the additional 3-year period, we estimate that 64–193 additional lives were saved, reducing the overall cost per life saved to US$2694–4382 ($702–1142 excluding solar). Translating lives saved directly to DALYs-averted equates to a cost of US$82–125 per DALY-averted ($22–35 excluding solar).

**Table 4:** Cost-effectiveness of improved hospital oxygen system for children (aged under 15 years, excluding neonates) with pneumonia during the original 2-year intervention and extrapolated to 5 years

Model	Effect size	Observed deaths			Estimated deaths		DALYs averted	Cost per			
		2-year*	3-year follow-up	Total (5-year)	Counterfactual†	Averted‡		DALY averted	Life saved	DALY averted (excl. solar)	Life saved (excl. solar)
Children U15		649	888	1537							
2-year*	0.50§	141	–	141	282	141	4653	$193.37	$6381	$50.37	$1662
5 year 100%¶	0.50§	141	193	334	668	334	11 020	$81.65	$2694	$21.27	$702
5 year 50%**	0.75††	141	193	334	539	205	6775	$132.80	$4382	$34.59	$1142

Costs expressed in US$ at the time expenditure (2015–2017).

*Restricted to the original 2-year intervention period.^26^

†Total multiplied by Effect size.

‡Counterfactual minus Total.

§Effect size using the estimate for full oxygen system compared to the pre-intervention period (OR 0.5, 95% CI 0.26 to 0.98).^26^

¶Extrapolated to include additional 3-year follow-up period with same effect size as observed during the original 2-year intervention period.

**Extrapolated to include additional 3-year follow-up period with attenuated effect size.

††Effect size using a 50% reduced effect estimate for full oxygen system compared to pre-intervention period applied to deaths during the follow-up period (0.75).

DALY, disability-adjusted life year.

Restricting this analysis to children with pneumonia aged under 5 years revealed similar cost-effectiveness estimates, suggesting that the greatest benefit is realised in younger children (who have higher pneumonia prevalence and mortality) (online supplemental table S7 Online Supplemental material).

#### Practical lessons

Feedback from hospital engineers, nurses and doctors and reflection on our programme experiences have provided lessons on how to sustainably improve oxygen practices, optimise the opportunities of renewable power technologies and strengthen medical equipment maintenance and management systems (table 5). While we based our approach on bedside oxygen concentrator technology and team-based quality improvement (similar to other oxygen improvement programmes in Africa and Asia-Pacific),^14 22 23 26 36 37^ many of these lessons are relevant irrespective of oxygen technology.

**Table 5:** Successful strategies and opportunities for improved implementation of improved hospital oxygen systems

Things that worked well	Ideas for improvement
Planning and engagement Early engagement of hospital managers and administrators, senior and junior doctors and nurses and biomedical engineers and technicians Comprehensive needs assessment to inform planning decisions Formation of multidisciplinary ‘oxygen teams’ to champion oxygen activities and navigate practical challenges	Planning and engagement More frequent and clearer interactions with local and state health leaders (eg, Commissioner for Health) to increase political commitment and translate learning into policy action More individualised oxygen packages to meet whole of hospital needs (paediatric, adult, emergency, surgery, etc.) and provide the most appropriate and affordable solutions
Clinical capacity building Practical, task-based training conducted onsite locally helping to translate learning into action immediately Regular mentoring visits and local project nurse to encourage and troubleshoot challenges	Clinical capacity building Refresher modules offered to facilities to make local retraining easier Improved integration with other training (eg, Emergency Triage Assessment and Treatment course), and orientation for new/rotating staff, to provide more holistic and sustainable capacity building
Equipment and maintenance Procurement of homogeneous equipment to enable easy and efficient training and repair Central hub for spare parts and repair workshop to ensure ready access for all hospitals Swap-and-go exchange of faulty equipment requiring major repairs to avoid excessive down-time Inclusion of biomedical engineers/technicians in decision-making teams to elevate their role and motivation	Equipment and maintenance Increased frequency of contact with hospital staff to encourage routine equipment care and early action to address faults Improved equipment tracking and maintenance information system to enable timely response and planning
Power supply Located a reliable local solar power provider to plan, instal and support hospitals	Power supply Consider whole-of-facility power supply improvements to improve overall power efficiency and affordability and diversify power options Develop ‘hybrid’ power systems, supplementing renewables such as solar with other sources, to improve affordability and reliability

## Discussion

Our analysis of follow-up data from a multisite hospital oxygen systems improvement programme shows that oxygen equipment and clinical practices can be sustained in the medium-term and that oxygen system improvements can be highly cost-effective.

### Clinical use

Overall, pulse oximetry and oxygen coverage rates were sustained at 5 year follow-up, with 9/12 facilities comfortably above the 80% coverage targets. Of the remaining facilities, two (H2 and H9) were small, rural hospitals that had previously achieved high coverage rates, and one (H7) was a large, urban hospital that had struggled to achieve high coverage even during the intensive intervention period.^26^ We previously found three interrelated mechanisms that supported pulse oximetry adoption: changed attitudes and motivation to view oximetry as a help (not more work); practical training that convinced people ‘why’ it mattered and positive support and role modelling from key influencers.^13^ Although we did not explore these explicitly in follow-up, reports from H7 HCWs identified: discontent between nursing, medical and managerial leaders; transition to user-pay financing system; loss of key positive influencers and relocation of key oxygen equipment to other departments. Changeover of leaders and loss of positive influencers was also reported from H2.

It is encouraging to see that oximetry and oxygen practices could be sustained without ongoing retraining or external intervention. Pulse oximetry has clearly demonstrated its utility, introduced as a fundamentally new practice in 11/12 hospitals and becoming a routine part of care with demonstrated investment by hospitals to replace broken oximeters. However, we should not underestimate the significant education and support during the early adoption period, particularly the targeted work with multidisciplinary teams to identify and respond to challenges. Other studies have shown that, while the training effects on clinical practice typically erode over time, ‘group problem solving’ approaches can deliver greater and more sustained improvements.^38 39^

### Equipment and maintenance

The COVID-19 pandemic resulted in an unprecedented demand for pulse oximeters and oxygen concentrators, highlighting the importance of pulse oximeters in all hospitals and the usefulness of concentrators for smaller facilities and emergency response.^40–43^ However, while oxygen concentrators and pulse oximeters are recognised as priority medical devices,^42 44^ commercially available items vary widely in cost, quality, maintenance requirements and warranty periods—with many not meeting technical specifications from WHO.^45 46^

Pulse oximeters have particular challenges when used for young children (who move and cry), severely unwell patients (with poor perfusion) and people with deeply pigmented skin tone.^47–49^ The quality of oximeters comes both from hardware (eg, robust probes that fit comfortably) and software (eg, computers that can account for low perfusion and darker skin tone). Most hospitals in our study invested in replacing broken oximeters but selected low-cost, finger-tip oximeters that may not be appropriate for severely unwell children in hospital.^47–49^

Oxygen concentrators have particular challenges when used in hot, humid and dusty environments and with low-quality power supply.^15 18 41^ An excellent study from The Gambia demonstrated that oxygen concentrators can function effectively for >6 years with regular care and maintenance provided by local staff and a centralised biomedical team.^15^ However, in practice, procurement of poorer quality concentrators and lack of technical capacity and coordination for repair has resulted in rapid failures.^18 41^ Notably, the hours of use from our programme concentrators (median 10 797 hours) was considerably higher than reported from the successful Gambian oxygen concentrator maintenance programme over a similar period (median 6267 hours; 1480 hours annually).^15^

In Nigeria’s decentralised hospital system, like many other contexts where procurement and management of medical equipment is the responsibility of individual facilities, equipment sustainability is a major challenge. Individual facilities varied in their technical capacity, with few having access to a biomedical engineer and no facilities operating equipment tracking or routine maintenance systems.^17^ Indeed, hospital technicians frequently told us that they only found out about many items of equipment when they were broken and needed repair. This reflects both the general undervaluing of technicians and biomedical capacity and the complexity of what is needed to effectively manage the range of medical equipment needed at even at a small hospital—from prioritisation, selection and procurement, to preventive and corrective maintenance, to quality assurance, documentation, and ongoing education.^50 51^

Our approach was to use tried and tested devices, upskill local healthcare workers and technicians on basic oxygen equipment care and maintenance and supplement this with additional support from a central hub (where the spare parts, tools and technical expertise were housed). We found that most concentrators were working well after many hours of use, but the condition and routine maintenance was patchy, and oximeters had almost all failed and required replacement. This suggests that, while the hub approach worked well during the first few years of frequent (3–6 monthly) visits, hospitals struggled with less intensive support in the latter years—particularly the remote facilities and those that experienced major structural or organisational change (eg, moving ward location, medical director change). This raises broader sustainability concerns relating to the gap in biomedical services available through existing government and private providers.

Like other oxygen systems improvement studies that have demonstrated mortality benefits, our approach used bedside oxygen concentrators to generate oxygen locally and supply multiple patients’ beds.^14 22 23 26 36 37^ Alternative oxygen sources include (i) pressure (or vacuum)-swing adsorption (PSA/VSA) plants that can be located onsite at the hospital or used to fill oxygen cylinders for distribution; and (ii) air-separation units producing pressurised liquid oxygen that can be used to fill liquid oxygen storage vessels onsite at facilities or depressurised and distributed in standard oxygen cylinders.^52^ These sources can cost-efficiently produce large volumes of oxygen and have a promising role not only in large, urban hospitals but also in extending supply chain capacity to smaller and more rural facilities—but they do come with additional technical and logistic requirements and challenges.^52^

### Solar power

Solar power is increasingly embraced as a reliable, affordable and sustainable power solution for health facilities in LMICs.^53^ Previous studies have reported successful deployment of solar power to run oxygen concentrators in diverse African and Asia-Pacific settings.^22 54–56^ Robust effectiveness and cost-effectiveness data are available from only one of these, a large-scale before-after trial in Papua New Guinea that reported a 60% reduction in child pneumonia and 40% reduction in all-cause child deaths at the cost of US$6435 per life saved.^22^

Most previous solar oxygen projects used international solar power providers, but we decided to use local providers. Our first installations resulted in complete failure and substantial time and financial loss due to a combination of inadequate design, low-quality components and lack of technician experience. Our subsequent installations were completed by a different local provider, with advice from international solar engineers. Staff and participating hospitals were generally pleased with the solar power installations and some facilities expanded and/or replaced solar-battery infrastructure. However, sustainability and cost were clear challenges, and our approach may not have been the most appropriate or cost-efficient power solution. We used stand-alone solar-battery systems to avoid the risks of integrating solar with lower quality power sources (eg, diesel generator, mains supply) and supplied power exclusively to the oxygen concentrators and ward lights (ie, no other equipment). However, this required large solar arrays and battery banks to ensure adequate power was available during periods of low solar yield, meant most power generated was not utilised and still required other power sources for additional equipment. Experience from other settings suggests that affordability and sustainability could be improved by a whole-of-facility approach that matched supply to aggregated power demand (thereby improving efficiency), and integration of solar with other power sources (so-called ‘hybrid’ solutions).^53^

### Cost-effectiveness

Our 2-year cost-effectiveness estimates (US$6381 per life saved; $193 per DALY-averted) are similar to estimates from Laos ($7289; $225)^14 24^ and Papua New Guinea ($1673; $50^23^ $6435; $195). Extrapolated over 5 years the cost-effectiveness of our hospital oxygen systems improvement programme ($2694–4382 per life saved, $82–133 per DALY-averted) ranks as a very cost-effective intervention, comparing favourably to established interventions already implemented at scale, for example, oral rehydration solution for diarrhoea (US$150/DALY-averted), artesunate treatment for severe malaria ($14–152/DALY-averted), essential programme for immunisation (<$100/DALY-averted), community management of severe acute malnutrition ($26–39/DALY-averted).^57 58^

Cost-effectiveness estimate should ideally represent *efficient* costs and expected benefits over the *full lifespan*.^59^ As such, we may have underestimated the true cost-effectiveness of improved hospital oxygen systems due to the inclusion of inefficient costs (eg, stand-alone solar power) and restriction of benefit to less than what may be expected over the full programme lifespan. We have also evaluated a relatively small-scale programme, and this will not capture the many opportunities to improve economies of scale and thereby increase cost-effectiveness.

We did not include costs recovered from patients in cost-effectiveness estimates. While some hospitals had started ‘revolving funds’ to direct money obtained for oxygen services back into oxygen system maintenance and expansion, most hospitals did not have a mechanism in place and were still charging patients in a very similar way as they had been doing at baseline.

### Limitations and generalisability

Our clinical data collection involved case note extraction, making it comparable to data reported in our previous study but was limited to 3 months so would not have captured seasonal variation in case-mix. However, we believe these limited clinical data were adequate to characterise oxygen and pulse oximetry practices and answer the primary question of how well practices were maintained after 4–5 years. Our hospital systems and technical data collection was obtained cross-sectionally using a standardised identification and testing approach but did rely on the recall and honesty of the key informants. We relied on routinely collected summary data to define admission and death numbers over the 3-year follow-up period and estimate cost-effectiveness. Due to lost records, we could not obtain this summary data from two small facilities, but these facilities contributed a small proportion of overall case and death numbers and would not have substantially influenced estimates.

We calculated costs based on actual expenditure in Naira (local costs) and USD (solar and other equipment orders), using the average 2016 exchange rate for conversion as this was when the bulk of costs were incurred. Nigeria experienced a nationwide recession between 2016 and 2020 with associated devaluation of the currency (most markedly in 2016).^28^ Devaluation means that local costs are relatively less expensive when expressed in USD, while international costs are relatively more expensive when expressed in Naira, compared to 2016. This would mean our reported costs, expressed in USD, are moderately higher than what would be incurred today. Conversely, equipment costs are currently much higher if using local currency than in 2016.

This study was conducted in sub-Saharan Africa’s malaria-endemic, lower-middle-income country context. Costs and benefits may differ in other contexts and when using different approaches to strengthening oxygen systems. However, we believe that the broad findings apply to many other low-income and middle-income contexts, particularly for small and rural hospitals that struggle to make oxygen services accessible to their population. This follow-up study was done during the COVID-19 pandemic and it is possible that the health system response may have influenced pulse oximetry and oxygen availability or use in participating hospitals. This study focused on children, but oxygen systems are clearly important for adolescents and adults, including for obstetric, surgical and critical care. Much less is known about the prevalence of hypoxaemia or the impact of improved oxygen systems on mortality in these older populations.^60^ Clearly, pulse oximetry is an important and low-cost priority for all acutely unwell, surgical and obstetric populations, and has been central to the safe surgery and essential critical care initiatives.^61–63^ Oxygen concentrator-based solutions have been successfully implemented in adult wards and operating theatres by ourselves and others, including for COVID-19 pandemic response.^43^ However, larger health facilities with higher adult critical care caseloads may be better served by higher volume oxygen sources such as PSA plants or liquid oxygen with high-pressure piping.^52^

Future research is needed into how to (i) improve hospital oxygen systems at scale (including larger oxygen production technology), (ii) integrate oxygen services across programmes (newborn/child and adult, pneumonia and other acute care^61^ and (iii) establish and finance delivery models that provide oxygen as a service (rather than oxygen equipment as a good).

## Conclusion

Hospital-level improvements to oxygen and pulse oximetry systems can be sustained over the medium-term and are a highly cost-effective child pneumonia intervention. Further improvements to effectiveness, sustainability and affordability may be possible with emerging technologies and broader investment in clinical and technical quality improvement systems.

## Data Availability

Data are available upon reasonable request. Anonymised clinical data are available on reasonable request to the corresponding author. All other data are fully available in the manuscript and supplemental material.
