# Supplementary material for: Cost-effectiveness and sustainability of improved hospital oxygen systems in Nigeria
Source: BMJ Glob Health. 2022 Aug 10;7(8):e009278. doi: 10.1136/bmjgh-2022-009278 (PMC9379491; doi:10.1136/bmjgh-2022-009278)
Supplement: Supplementary data [file bmjgh-2022-009278supp001.pdf]

Reflexivity Statement

Graham HR, Bakare AA, Ayede AI, et al. Cost-effectiveness and sustainability of improved hospital oxygen systems in Nigeria. BMJ global health.

Study conceptualisation	1. How does this study address local research and policy priorities?<br>This study is part of a broader programme implementation research programme seeking better ways to improve hospital oxygen systems – a priority for low- and middle-income countries globally, especially in the African region. The programme was conceived and implemented in partnership with local stakeholders, including hospital management and state government. Our Nigeria and international programme team members continue to participate in local, state, and national activities translating research findings into practice, including as founding members of the United for Oxygen coalition convened by the Federal Ministry of Health.<br>2. How were local researchers involved in study design?<br>AGF, AIA, and AAB led the original Oxygen Implementation programme, including every step of the study design and conduct, from developing research questions and meeting with health facilities to implementation, data collection, write-up, and dissemination. JE, OO, OB, and EB led the data collection and had input into revising data collection instruments and processes. AGF and AAB are core members of the national United for Oxygen coalition and the global Every Breath Counts coalition and are recognised as thought leaders in this area.
Research management	1. How has funding been used to support the local research team(s)?<br>This programme of work was funded by a grant from the Bill and Melinda Gates Foundation (BMGF), with a subgrant to University College Hospital (UCH) Ibadan (Prof A.G. Falade). The vast majority (>90%) of staff were employed locally through UCH, including several junior researchers and ~60% female staff, including work towards postgraduate degrees.
Data acquisition and analysis	1. How are research staff who conducted data collection acknowledged?<br>Research staff who conducted data collection are acknowledged as co-authors (where they meet authorship criteria) or in the Acknowledgements (by name where approval was given).<br>2. How have members of the research partnership been provided with access to study data?

	All members of the partnership have access to data. A full copy of all data relating to the project is retained at the local site (UCH), with a secondary copy and most cleaning/analysis performed at the international site (MCRI).<br>3. How were data used to develop analytical skills within the partnership?<br>Nigeria staff have used data from this work programme for research team training and individual thesis/projects. The original programme manager is now pursuing doctoral studies internationally under the supervision of the local primary investigator (AGF) and international programme leader (HRG).
Data interpretation	1. How have research partners collaborated in interpreting study data?<br>The data for this study was supplemented by reflections from the local clinical and technical study staff from the data collection point. Early draft results were shared with the lead local investigators and data collectors for checking and interpretation. The key table from the Results section is one of the unique products of our joint interpretation of results.
Drafting and revising for intellectual content	1. How were research partners supported to develop writing skills?<br>Most of the writing for this study was done by HRG. Other Nigeria team members have led other papers and conference abstracts.<br>2. How will research products be shared to address local needs?<br>The results from this study will be shared through local and national forums, including the Nigeria United for Oxygen coalition – and internationally through the, Every Breath Counts coalition. These findings will also be shared through the local NGO Oxygen for Life Initiative, which works closely with hospitals and the state government to improve oxygen access.
Authorship	1. How is the leadership, contribution and ownership of this work by LMIC researchers recognised within the authorship?<br>The senior author is AGF – the Nigeria programme leader. Authors 2-6 include the local programme managers and data collectors who coordinated the follow-up data collection. Other authors represent senior academics and technical experts who provided oversight and supervision.<br>2. How have early career researchers across the partnership been included within the authorship team?<br>The first author is a junior post-doc who completed their doctoral work on the Nigeria Oxygen Implementation project. The second author is the programme

		manager, completing their doctoral research on a separate project. Authors 3-6 are non-academic clinicians and technicians.
	3.	How has gender balance been addressed within the authorship?<br>Six authors are female (OB, EB, EN, BM, AG, AIA), and nine are male (HRG, AAB, JE, OO, SQ, RI, DP, TD, AGF). The preponderance of males is partly due to the paucity of female biomedical engineering staff in Nigeria (and globally) and partly because all external organisational representatives were male (SQ, RI, DP).
Training	1.	How has the project contributed to training of LMIC researchers?<br>This work programme included both formal training (programme management, protocol development, ethics, etc.) and a supportive feedback environment for on-the-job learning. This programme of work has launched the academic career of one early-career researcher from (currently doing PhD), given junior staff their first insight into research, and advanced the career of two other mid- and senior-career researchers.
Infrastructure	1.	How has the project contributed to improvements in local infrastructure?<br>This study outlines how this programme directly benefited 12 secondary-level hospitals and the degree to which this support was sustained over time. It had secondary effects on ~12 additional hospitals not included in the study but benefited from improved hospital oxygen systems. We leveraged additional donor support to expand these services early in the COVID19 pandemic for child and adult hospital care.
Governance	1.	What safeguarding procedures were used to protect local study participants and researchers?<br>This study did not involve direct patient contact and limited access to clinical records for quality improvement audits. Data collectors were all trained and used standard procedures developed during our previous work. We followed institutional guidance on staff safety, including road travel and COVID-19 precautions – with substantial contact with facilities conducted remotely. We adapted MCRI procedures for child protection and participant photography to supplement UCH institutional procedures.

Graham HR, *et al. BMJ Global Health* 2022; 7:e009278. doi: 10.1136/bmjgh-2022-009278
