# Supplementary material for: Cost-effectiveness and sustainability of improved hospital oxygen systems in Nigeria
Source: BMJ Glob Health. 2022 Aug 10;7(8):e009278. doi: 10.1136/bmjgh-2022-009278 (PMC9379491; doi:10.1136/bmjgh-2022-009278)
Supplement: Supplementary data [file bmjgh-2022-009278supp002.pdf]

Supplemental material

Graham HR, Bakare AA, Ayede AI, et al. Cost-effectiveness and sustainability of improved hospital oxygen systems in Nigeria. BMJ global health.

Contents

Supplemental material...1
Table S1 - Facility characteristics (Follow up) (EXTENDED) 2
Table S2 – Facility characteristics (Baseline)...3
Table S3 – Admissions and Deaths...4
Table S4 – Effect of the intervention on practice outcomes (mixed-effects model)...6
Figure S1 – Heat map of practice outcomes by facility...8
Table S5 – Concentrator test results...9
Table S6 – Program costs...10
Table S7 – Cost effectiveness...11

Graham HR, *et al. BMJ Global Health* 2022; 7:e009278. doi: 10.1136/bmjgh-2022-009278

Table S1 - Facility characteristics (Follow up) (EXTENDED)

Facility characteristics at 12 secondary-level hospitals in south-west Nigeria on follow up assessment in 2021.

Hospital	H1	H2	H3	H4	H5	H6	H7	H8	H9	H10	H11	H12
**Hospital Type**	Mission	Mission	State	State	State	Mission	State	State	State	Mission	Mission	State
**Paediatric Beds (child + neonatal)**	64 (34+30)	29 (21+8)	23 (18+5)	47 (20+27)	36 (16+20)	25 (25+0)	37 (15+22)	35 (22+13)	11 (11+0)	47 (34+13)	12 (12+0)	26 (26+0)
**Mean admissions/month[a]**												
**Child**	108	?[e]	70	186	55	24	186	41	?[e]	92	24	45
**Neonate**	71	?[e]	7	48	14	NA	34	15	NA	18	NA	NA
**Consultant access[b]**	Yes	Yes	Yes	Yes	Yes	Yes	Yes	Yes	Yes	Yes	Yes	No
**Doctors - paediatrics**	3	8	3	2	1	4	4	8	2	2	4	1
**Nurses - paediatrics**	8	7	14	10	10	6	15	14	10	7	3	8
**Per shift (AM/PM/Night)**	3/2/2	1/1/1	2/-/2	2/2/2	3/2/1	2/-/2	3/2/2	4/2/2	2/1/1	2/-/1	1/-/1	2/2/1
**Nurses – neonates[c]**	13	6	14	12	11	NA	15	11	NA	6[b]	NA	NA
**Per shift (AM/PM/Night)**	3/2/2	3/1/1	2/-/2	2/2/2	3/1/1		4/4/4	2/2/2		2/-/1		
**Power (hours/day)**												
Paediatric ward	24	11	24	18	7	24	8	15	18	12	16	8
Neonatal ward	24	11	24	18	7	-	8	15	-	16	-	-
**Admission register**	Yes	Yes	Yes	Yes	Yes	Yes	Yes	Yes	Yes	Yes	Yes	Yes
**Individual patient notes**	Yes	Yes	Yes	Yes	Yes	Yes	Yes	Yes	Yes	Yes	Yes	Yes
**Medical education**	Yes	No	Yes	No	Yes	Yes	No	No	Yes	No	No	Yes
**Nursing education**	Yes	No	Yes	No	Yes	No	No	No	Yes	No	No	Yes
**Clinical guidelines**	Yes	Yes	Yes	No	Yes	No	No	Yes	Yes	Yes	Yes	Yes
**M&M meetings**	No[d]	No[d]	No[d]	No[d]	Yes	Yes	No[d]	No[d]	No	No[d]	No[d]	No[d]

Notes: Neonate ≤28 days, Child 29 days-15 years; M&M = morbidity and mortality; NA = not applicable. (a) Mean monthly admissions for three years, January 2018 to December 2020 inclusive; (b) Family Medicine or Paediatric Consultant; (c) Included two midwives; (d) Hospital-wide M&M meetings but no paediatric meeting; (e) missing data due to lost admission books. H6, H9, H11, and H12 do not admit neonates

Table S2 – Facility characteristics (Baseline)

Facility characteristics at 12 secondary-level hospitals in south-west Nigeria at baseline in 2015.

**Hospital**	**H1**	**H2**	**H3**	**H4**	**H5**	**H6**	**H7**	**H8**	**H9**	**H10**	**H11**	**H12**
**Hospital Type**	Mission	Mission	State	State	State	Mission	State	State	State	Mission	Mission	State
**Paediatric Beds (child + neonatal)**	**70** (40+30)	**32** (20+12)	**25** (21+4)	**36** (16+20)	**60** (44+16)	**20** (15+5)	**48** (20+28)	**46** (22+24)	**13** (9+4)	**63** (38+25)	**14** (12+2)	**36** (26+10)
**Mean admissions / month**												
**Child**	**86**	**32**	**65**	**85**	**69**	**29**	**133**	**46**	**12**	**109**	**25**	**24**
**Neonate**	**103**	**4**	**6**	**78**	**12**	**8**	**89**	**26**	**0**	**61**	**1**	**0**
**Admissions, Jan 14-Dec 15**	**4530**	**863**	**1696**	**3908**	**1655**	**699**	**5327**	**1721**	**284**	**4087**	**637**	**588**
**Child**	2056	772	1555	2028	1357	488	3181	1104	281	2612	602	587
**Neonate**	2474	91	141	1880	298	211	2146	617	3	1475	35	1
**Hospital Staffing**												
**Consultant access**	Yes	Yes[b]	Yes	Yes	Yes	Yes	Yes	No	Yes[b]	Yes	Yes	No[b]
**Doctors - entire hospital**	4	4	2	11	17	5	16	12	7	6	6	7
**Nurses - child/newborn wards (#paed trained)**	18	7	16 (2)	33 (3)	62	9 (2)	26	31	11	18	4	26
**Hospital Oxygen Supply**												
**Oxygen cylinders**	Yes[d]	Yes[d]	Yes[d]	Yes	Yes	Yes[d]	Yes[c]	Yes	Yes[d]	Yes	Yes[d]	Yes[d]
**Oxygen concentrators[v]**	Yes[e]	Yes[e]	Yes[e]	Yes[e]	No	Yes	Yes[e]	No	No	Yes[e]	Yes[e]	No
**Power (hours/day)**	12-18	6-12	>18	6-12	6-12	6-12	12-18	6-12	12-18	6-12	12-18	>18
**Pulse oximeters [f]**	0	0	0	0	3	1	0	0	0	0	1	0
**Oxygen delivery devices**												
**Nasal prongs**	Yes	No	Yes	Yes	Few	Few	Few	Few	Few	Yes	Yes	Few
**Nasal catheters**	Yes	Yes	Yes	Yes	Yes	Few	No	Yes	No	Few	No	No
**Washed and re-used**	Rarely	No	Yes	Yes	Yes	Yes	No	Yes	No	Yes	Yes	Yes
**Oxygen cost (per day) [g]**	N1000	N12000	N1500	N4000	N3500	N9600	FREE	N3600	N7500	N19200	N6000	N2400
- **USD**	$5.43	$65.22	$8.15	$21.74	$19.02	$52.17		$19.57	$40.76	$104.35	$32.61	$13.04

Notes: Neonate ≤28 days, Child 29 days-15 years. (a) Mean monthly admissions for two years, January 2014 to December 2015; (b) Family Medicine Consultant; (c) piped system connected to large oxygen cylinder; (d) not available in paediatric areas; (e) present but not fit for use; (f) H6 was the only hospital routinely practising pulse oximetry; (g) Average daily cost per patient (individual hospitals variably charged per hour, per day, per patient, per cylinder), with conversion to USD at 1 January 2015 exchange rate (184:1)

Table S3 – Admissions and Deaths

Estimated number of child (aged under 15 years, excluding neonates) ADMISSIONS to 12 hospitals during pre-intervention (2014-2015), intervention (2016-2017), and follow up periods (2018-2020).

Hospital	2-year pre-intervention	2-year intervention	3-year extrapolated	3-year follow-up	Variance
H1	1882	1987	2980.5	3892	131%
H2	714	593	889.5	*889.5*	-
H3	1381	1620	2430	2525	104%
H4	1931	1702	2553	2596	102%
H5	1207	1835	2752.5	1975	72%
H6	469	544	816	868	106%
H7	2896	2897	4345.5	6702	154%
H8	969	1380	2070	1478	71%
H9	245	509	763.5	*763.5*	-
H10	2521	2236	3354	3295	98%
H11	561	872	1308	860	66%
H12	528	968	1452	1607	111%
**TOTAL**	**15304**	**17143**	**25714.5**	**27450**	**107%**
TOTAL excluding two hospitals with no summary follow-up data				25797	**107%**

Three-year extrapolation from the 2-year intervention period (i.e. *3/2); Admission and death data unavailable at H2 and H9; Variance expressed as 3-year documented follow-up numbers as a proportion of 3-year extrapolated.

Estimated number of child (aged under 15 years, excluding neonates) DEATHS at 12 hospitals during pre-intervention (2014-2015), intervention (2016-2017), and follow up periods (2018-2020).

Hospital	2-year pre-intervention	2-year intervention	3-year extrapolated	3-year follow-up	Variance
H1	130	101	151.5	152	100%
H2	51	37	55.5	*55.5*	-
H3	88	122	183	73	40%
H4	79	70	105	79	75%
H5	28	59	88.5	55	62%
H6	29	21	31.5	29	92%
H7	49	70	105	197	188%
H8	32	75	112.5	48	43%
H9	4	19	28.5	*28.5*	-
H10	105	86	129	116	90%
H11	29	32	48	21	44%
H12	8	26	39	34	87%
**TOTAL**	**632**	**718**	**1077**	**888**	**82%**
TOTAL excluding two hospitals with no summary follow-up data				804	**81%**

Three-year extrapolation from the 2-year intervention period (i.e. *3/2); Admission and death data unavailable at H2 and H9; Variance expressed as 3-year documented follow-up numbers as a proportion of 3-year extrapolated.

Graham HR, *et al. BMJ Global Health* 2022; 7:e009278. doi: 10.1136/bmjgh-2022-009278

Observed and estimated number of neonates and children admitted and receiving oxygen at 12 secondary hospitals in Nigeria.

NEONATES	2-year pre-intervention	2-year intervention	3-month 2021	3-year follow-up
**Total admissions**	**7934**	**7638**	**392**	**5412**
Number receiving oxygen	2029	2429	113	1560
Proportion receiving oxygen	26%	32%	29%	29%
**CHILDREN U15**	**2-year pre-intervention**	**2-year intervention**	**3-month 2021**	**3-year followup**
**Total admissions**	**12782**	**16928**	**628**	**274450**
Number receiving oxygen	1468	2257	88	3846
Proportion receiving oxygen	11%	13%	14%	14%

Three-year follow-up numbers derived from observed admission numbers (from ward admission registers) and proportion receiving oxygen extrapolated from observed 3-month follow-up data.

Graham HR, *et al. BMJ Global Health* 2022; 7:e009278. doi: 10.1136/bmjgh-2022-009278

Table S4 – Effect of the intervention on practice outcomes (mixed-effects model)

Effect of the intervention(s) on practice outcomes, comparing pulse oximetry, full oxygen system, and follow-up periods to the preintervention period.

Population, study period	Proportions	Mixed-Model Adjusted Odds Ratio (95% CI)$^{\alpha}$		ICC
	n/N* (%)			**(95% CI)**
**Neonates**			***p*-Value**	
Pulse oximetry performed				
Preintervention	219/7,940 (2.8%)	-		
Pulse oximetry	1,723/2,569 (67.1%)	18,543 (1,277-269,217	<0.001	0.64
Full O2 system	4,122/4,367 (94.4%)	90,687 (9,460-869,372)	<0.001	(0.43-0.81)
Follow up	326/392 (83.2%)	12,446 (1,331-116,300)	<0.001	
Oxygen provided				
Preintervention	1,794/7,940 (22.6%)	-		
Pulse oximetry	559/2,569 (21.8%)	1.27 (0.81-2.00)	0.298	0.06
Full O2 system	1,416/4,367 (32.4%)	2.04 (1.46-2.83)	<0.001	(0.02-0.15)
Follow up	113/392 (28.8%)	1.52 (1.04-2.23)	0.032	
Oxygen provided if SpO_2 < 90%				
Preintervention	24/30 (80.0%)	-		
Pulse oximetry	307/380 (80.8%)	1.83 (0.18-18.4)	0.609	0.02
Full O2 system	833/921 (90.4%)	1.49 (0.17-13.3)	0.719	(0.00-0.19)
Follow up	50/53 (94.3%)	2.64 (0.23-30.9)	0.44	
Oxygen provided if child had WHO danger signs				
Preintervention	757/1206 (62.8%)	-		
Pulse oximetry	288/523 (55.1%)	0.50 (0.24-1.07)	0.074	0.04
Full O2 system	600/830 (72.3%)	1.10 (0.64-1.91)	0.729	(0.01-0.11)
Follow up	33/50 (66.0%)	1.13 (0.51-2.52)	0.758	
Had clinical indication for oxygen if oxygen provided[¥]				
Preintervention	764/1,794 (42.6%)	-		
Pulse oximetry	424/559 (75.9%)	6.03 (2.70-14.0)	<0.001	0.08
Full O2 system	1041/1416 (73.5%)	4.75 (2.65-8.52)	<0.001	(0.03-0.21)
Follow up	67/113 (59.3%)	2.11 (1.07-4.17)	0.032	
**Children**				
Pulse oximetry performed				
Preintervention	500/12,737 (3.9%)	-		
Pulse oximetry	3,941/6,298 (62.6%)	4,266 (219-82,815)	<0.001	0.29
Full O2 system	8,463/9,216 (91.8%)	23,530 (1,983-279,138)	<0.001	(0.16-0.47)
Follow up	506/628 (80.6%)	11,424 (919-142,080)	<0.001	
Oxygen provided				

Preintervention	1,184/12,737 (9.3%)	-		
Pulse oximetry	455/6,298 (7.2%)	0.72 (0.44-1.19)	0.196	0.14
Full O2 system	1,262/9,216 (13.7%)	1.50 (1.04-2.15)	0.028	(0.06-0.28)
Follow up	88/628 (14.0%)	1.48 (0.97-2.28)	0.071	
Oxygen provided if SpO_2 < 90%				
Preintervention	34/46 (73.9%)	-		
Pulse oximetry	265/395 (67.1%)	3.55 (0.23-54.9)	0.364	0.15
Full O2 system	694/851 (81.6%)	7.87 (0.59-105.4)	0.119	(0.05-0.37)
Follow up	50/57 (87.7%)	14.6 (0.92-232.6)	0.058	
Oxygen provided if child had WHO danger signs				
Preintervention	745/4,489 (16.6%)	-		
Pulse oximetry	328/2291 (14.3%)	0.75 (0.42-1.36)	0.347	0.09
Full O2 system	757/3024 (25.0%)	1.49 (0.97-2.28)	0.069	(0.03-0.20)
Follow up	36/226 (15.9%)	0.78 (0.45-1.36)	0.379	
Had indication for oxygen if oxygen provided[¥]				
Preintervention	753/1,184 (63.6%)	-		
Pulse oximetry	397/455 (87.3%)	2.75 (1.30-5.81)	0.008	0.17
Full O2 system	1,045/1,262 (82.8%)	2.12 (1.28-3.51)	0.003	(0.07-0.38)
Follow up	64/88 (72.7%)	1.48 (0.76-2.88)	0.25	

Data are *n*/*N* (%) unless otherwise indicated.

*Denominators vary according to the population included.

[α]Mixed-model odds ratios account for the clustering of patients within hospitals and adjust for time trends. Under the stepped-wedge design, the adjusted odds ratios are calculated using all data points in the intervention period versus the comparison period and therefore represent the average odds of exposure to the intervention.

[¥]'Indication for oxygen' is calculated as [# with SpO_2 < 90% or WHO emergency signs on admission)/# prescribed oxygen therapy]. This outcome measure may be biased given the dramatic change in SpO2 documentation over the study periods.

Abbreviations: CI, confidence interval; ICC, intracluster correlation coefficient

Figure S1 – Heat map of practice outcomes by facility

Care practice outcome rates (proportions), by step and hospital, including follow-up (FU) period.

Steps (4-month periods)

A Neonates: Pulse oximetry performed (n=15971)

Hospitals	1	2	3	4	5	6	7	8	9	10	11	12	FU
1	0%	0%	0%	0%	0%	0%	56%	98%	99%	99%	99%	96%	98%
2	0%	0%	0%	0%	0%	7%	56%	86%	38%	100%	100%	93%	0%
3	0%	0%	0%	0%	0%	0%	73%	100%	100%	100%	100%	100%	93%
4	0%	0%	0%	0%	0%	0%	57%	66%	85%	75%	84%	98%	100%
5	0%	0%	0%	0%	0%	0%	56%	98%	100%	100%	100%	100%	97%
6	95%	87%	85%	96%	97%	93%	100%	100%	100%	100%	94%	100%	
7	0%	0%	0%	0%	0%	4%	46%	47%	45%	91%	91%	82%	0%
8	0%	0%	0%	0%	0%	1%	48%	63%	98%	99%	100%	98%	97%
9		0%					0%	0%	100%	100%	100%		
10	0%	0%	0%	1%	0%	7%	76%	94%	94%	98%	97%	100%	99%
11	0%	0%	0%	0%	0%	0%	33%	55%	88%	77%	100%	100%	100%
12													

B Children: Pulse oximetry performed (n=30260)

Hospitals	1	2	3	4	5	6	7	8	9	10	11	12	FU
1	0%	0%	1%	0%	0%	0%	79%	95%	94%	98%	88%	85%	98%
2	0%	0%	0%	0%	1%	1%	47%	92%	88%	98%	97%	98%	16%
3	0%	0%	0%	0%	0%	0%	35%	99%	100%	100%	100%	99%	99%
4	0%	0%	1%	0%	0%	6%	66%	78%	87%	90%	97%	88%	96%
5	0%	0%	0%	0%	0%	0%	53%	90%	100%	100%	100%	91%	96%
6	98%	93%	79%	93%	97%	97%	99%	95%	90%	98%	98%	94%	100%
7	0%	0%	0%	0%	0%	2%	37%	34%	51%	93%	72%	51%	0%
8	0%	0%	0%	0%	0%	0%	54%	54%	46%	88%	99%	98%	95%
9	0%	0%	0%	0%	0%	0%	30%	69%	29%	92%	100%	90%	44%
10	0%	0%	0%	0%	0%	2%	57%	89%	91%	99%	97%	99%	89%
11	0%	4%	0%	0%	17%	28%	84%	99%	98%	96%	95%	97%	96%
12	0%	0%	0%	0%	0%	0%	55%	61%	13%	55%	96%	93%	89%

C Neonates: Oxygen provided (n=15971)

Hospitals	1	2	3	4	5	6	7	8	9	10	11	12	FU
1	28%	27%	24%	27%	26%	24%	26%	36%	37%	42%	40%	40%	39%
2	0%	12%	36%	13%	25%	27%	38%	41%	44%	58%	55%	53%	0%
3	33%	23%	50%	46%	47%	10%	23%	24%	56%	11%	43%	81%	29%
4	12%	15%	21%	13%	15%	18%	12%	14%	29%	22%	24%	21%	27%
5	13%	25%	4%	0%	15%	14%	7%	9%	27%	14%	25%	29%	5%
6	16%	53%	44%	31%	44%	64%	23%	52%	54%	44%	67%	78%	
7	30%	24%	32%	21%	18%	31%	29%	29%	31%	28%	26%	24%	20%
8	12%	32%	45%	21%	20%	27%	21%	13%	24%	21%	24%	32%	32%
9		0%					0%	0%	100%	0%	0%		
10	22%	18%	18%	26%	18%	20%	19%	16%	18%	24%	29%	31%	31%
11	33%	100%	0%	0%	15%	0%	0%	9%	8%	0%	10%	32%	67%
12													

D Children: Oxygen provided (n=30260)

Hospitals	1	2	3	4	5	6	7	8	9	10	11	12	FU
1	13%	9%	14%	9%	11%	12%	11%	18%	24%	20%	19%	22%	23%
2	2%	8%	9%	6%	9%	7%	5%	12%	21%	10%	14%	9%	9%
3	12%	9%	8%	10%	20%	12%	16%	24%	18%	20%	20%	23%	20%
4	11%	8%	19%	7%	12%	17%	5%	9%	14%	8%	9%	11%	16%
5	11%	4%	1%	2%	4%	2%	3%	0%	10%	13%	10%	12%	15%
6	19%	20%	16%	8%	12%	11%	5%	18%	19%	15%	7%	11%	0%
7	6%	10%	16%	9%	9%	12%	6%	10%	11%	8%	4%	13%	7%
8	21%	15%	15%	6%	12%	12%	6%	11%	8%	16%	13%	12%	5%
9	0%	0%	0%	0%	0%	0%	0%	0%	0%	3%	1%	3%	0%
10	8%	6%	5%	8%	9%	11%	6%	8%	9%	7%	12%	16%	22%
11	9%	6%	8%	5%	24%	10%	10%	7%	8%	6%	7%	10%	10%
12	0%	0%	0%	0%	0%	2%	0%	1%	1%	0%	8%	8%	7%

E Neonates: Oxygen provided, if SpO2<90% (n=1501)

Hospitals	1	2	3	4	5	6	7	8	9	10	11	12	FU
1	0%				0%		75%	87%	95%	87%	91%	87%	89%
2						100%	80%	80%	100%	86%	100%	100%	
3							33%	60%	100%	0%	100%	100%	100%
4							53%	71%	89%	76%	97%	97%	75%
5							100%	75%	80%	89%	100%	95%	100%
6			75%	83%	100%	100%	67%	86%	100%	75%	83%	90%	
7						100%	85%	90%	98%	100%	94%	91%	
8						100%	62%	80%	83%	89%	100%	95%	100%
9									100%				
10						67%	85%	80%	100%	93%	90%	86%	100%
11							0%		100%		50%	67%	100%
12													

F Children: Oxygen provided, if SpO2<90% (n=1455)

Hospitals	1	2	3	4	5	6	7	8	9	10	11	12	FU
1			100%	100%			85%	93%	84%	91%	85%	83%	100%
2						100%	11%	56%	82%	58%	83%	45%	100%
3							79%	64%	88%	96%	65%	81%	94%
4			0%			100%	40%	94%	85%	60%	81%	85%	100%
5							50%	10%	79%	87%	94%	94%	100%
6	71%	75%	100%	71%	100%	100%	100%	100%	100%	100%	67%	100%	
7							50%	81%	86%	93%	88%	100%	
8		0%					58%	82%	86%	87%	76%	73%	100%
9							0%	0%	0%	25%	100%	100%	0%
10						0%	71%	78%	82%	67%	83%	80%	80%
11					100%	33%	90%	67%	100%	45%	86%	76%	100%
12							0%	29%	0%	0%	79%	70%	50%

Each cell contains the proportion of patients who received a particular element of care (pulse oximetry or oxygen therapy) on admission. For each panel in this figure, the 12 hospitals are on the y-axis, and the trial steps are on the x-axis. The colour gradient extends from red (0%) through yellow to green (100%), providing a visual representation of practice change over time. When the denominator to compute the cell rate is 0, cells are coloured in white.

Graham HR, *et al. BMJ Global Health* 2022; 7:e009278. doi: 10.1136/bmjgh-2022-009278

Table S5 – Concentrator test results

Summary of the condition, functionality, and hours of use of 29 oxygen concentrators tested after 4-5 years of programme implementation

Location	Checklist[1]	Condition	Hours	Oxygen purity (%)	
				2LPM	5LPM (max)
H1 neonate	Poor	Moderate	1318.1	95.6	96.3
H1 neonate	Poor	Moderate	23000	95.5	92.7
H1 neonate	Poor	Moderate	32000	91.8	80.2-90.0
H1 child	Poor	Moderate	21320	95.2	96.1
H1 child	Poor	Moderate	21610	93.5	94
H1 child	Poor	Moderate	3636.5	95.9	96.5
H2 neonate	Missing	Good	?	90.7	90.1
H2 child	Missing	Good	6582.4	30.1	30.6
H3 child	Poor	Good	2163.4	24.3	25.4
H3 child	Poor	Good	?	86.2	86.7
H4 neonate	Poor	Good	10797	95.4	96.6
H4 neonate	Poor	Moderate	15235	98.9	97.5
H4 child	Good	Moderate	16809	95.4	97.2
H5 neonate	Good	Good	8201.7	93.3	94.1
H5 child	Good	Good	7448.7	40.6	40.36
H5 OPD	Good	Good	8203.9	93.4	92.5
H6	Missing				
H7 neonate	Poor	Good	21241	94	96.7
H7 neonate	Poor	Good	27251	95.2	97.6
H7 ED	Poor	Moderate	23674	94	96.9
H7	Missing				
H8 neonate	Poor	Good	15634	93.7	94.2
H8 child	Moderate	Poor	86624	92.7	93.7
H9 child	Missing	Moderate	473	20.6	20.6
H10 neonate	Good	Good	8868.4	93.7	94
H10 child	Missing	Good	16286	93.8	94
H10 child	Missing	Good	17447	93.7	94
H11 child	Missing	Good	4332.2	95.4	96.2
H12 child	Good	Good	632.3	91.7	85.7

Three additional concentrators were retired from service due to major faults (n=3). 1 – paper checklist attached to concentrator to guide nurses' routine (weekly) equipment checks.

ED, emergency department; LPM, litres per minute oxygen flow rate.

Table S6 – Program costs

Equipment, solar power, and programme implementation costs

	Cost (USD)	Quantity	Total cost	Per facility
**Equipment**			**$ 150,365**	**$ 12,530**
Oxygen concentrator 5LPM (Airsep)	$ 675	38	$ 25,650	$ 2,138
Flowmeter assembly (Airsep)	$ 960	30	$ 28,800	$ 2,400
Pulse oximeter + spare probes (Lifebox)	$ 270	79	$ 21,300	$ 1,775
Installation materials	$ 24,215	1	$ 24,215	$ 2,018
Spare parts	$ 10,000	1	$ 10,000	$ 833
Tools	$ 6,500	1	$ 6,500	$ 542
Nasal prongs	$ 3	1500	$ 3,900	$ 325
Shipping and customs	$ 30,000	1	$ 30,000	$ 2,500
**Solar power**			**$ 665,369**	**$ 55,447**
**Programme costs**			**$ 84,000**	**$ 7,000**
Installation and training	$ 5,000	12	$ 60,000	$ 5,000
Support and maintenance	$ 2,000	12	$ 24,000	$ 2,000
		**TOTAL**	**$ 899,734**	**$ 74,978**
		**Total without solar**	**$ 234,365**	**$ 19,530**

Costs are expressed in USD at the time of purchase (2015-2017). Spare parts included filters, sieve beds, compressors, printed circuit boards (PCBs), compressor repair kit, flowmeters, check valves, solenoids, tubing. Tools included oxygen analysers, installation, and repair tools.

Table S7 – Cost effectiveness

Cost-effectiveness of improved hospital oxygen system for children aged under 5 years (U5) and under 15 years (U15) (excluding neonates) with pneumonia during the original 2-year intervention and extrapolated to 5 years.

	Effect size	Deaths					DALYs averted	Cost per			
		2-year intervention	3-year follow-up	Total (5-year)	Est. counterfactual	Est. deaths averted		DALY averted	Life saved	DALY averted (excl. solar)	Life saved (excl. solar)
Children U15		*649*	*888*	*1,537*							
**2-year original** [a]	0.50 [d]	141	-	141	282	141	4,653	$ 193.37	$ 6,381	$ 50.37	$ 1,662
**5-year 100%** [b]	0.50 [d]	141	193	334	668	334	11,020	$ 81.65	$ 2,694	$ 21.27	$ 702
**5-year 50%** [c]	0.75 [e]	141	193	334	539	205	6,775	$ 132.80	$ 4,382	$ 34.59	$ 1,142
Children U5		*529*	*728*	*1,257*							
**2-year original** [a]	0.46 [d]	128	-	128	278	150	4,959	$ 181.45	$ 5,988	$ 47.26	$ 1,560
**5-year 100%** [b]	0.46 [d]	128	175	303	658	355	11,728	$ 76.71	$ 2,532	$ 19.98	$ 659
**5-year 50%** [c]	0.73 [e]	128	175	303	518	215	7,092	$ 126.87	$ 4,187	$ 33.05	$ 1,091

Costs expressed in US dollars at the time expenditure (2015-2017); (a) Restricted to the original 2-year intervention period[23]; (b) Extrapolated to include additional 3-year follow-up period with same effect size as observed during the original 2-year intervention period; (c) Extrapolated to include additional 3-year follow-up period with attenuated effect size; (d) Effect size using the estimate for full oxygen system compared to the pre-intervention period (U5: OR 0.46, 95% CI 0.23-0.92. U15: OR 0.5, 95% CI 0.26-0.98)[23]; (e) Effect size using a 50% reduced effect estimate for full oxygen system compared to pre-intervention period applied to deaths during the follow-up period (U5: OR 0.73. U15: 0.75).

Graham HR, *et al. BMJ Global Health* 2022; 7:e009278. doi: 10.1136/bmjgh-2022-009278
